# Social isolation induces hyperactivity and exploration in aged female mice

**DOI:** 10.1371/journal.pone.0245355

**Published:** 2021-02-03

**Authors:** D. Gregory Sullens, Kayla Gilley, Kendall Jensen, Elisabeth Vichaya, Sara L. Dolan, Melanie J. Sekeres

**Affiliations:** 1 Department of Psychology and Neuroscience, Baylor University, Waco, Texas, United States of America; 2 School of Psychology, University of Ottawa, Ottawa, Ontario, Canada; Technion Israel Institute of Technology, ISRAEL

## Abstract

Prolonged social isolation is associated with poor physical and mental health outcomes, findings observed in both humans, and rodent models of isolation. Humans, like mice, may engage in enhanced exploratory and social behaviour following isolation, which may protect against subsequent cognitive decline and psychological distress. Understanding how these effects may impact behaviour in older adults is particularly relevant, as this population is likely to experience periods of late-life social isolation. We report that late-life social isolation in female mice did not lead to robust depressive-like symptomology, altered social interaction behaviour, sensitivity to context fear acquisition and memory, or alterations in inflammatory cytokines (*IL-6*, *IL-1β*, *Tnf-α)* or microglial activation *(Itgam)* within the hippocampus. Rather, isolation increased hyperactivity and exploration behaviours. These findings have translational value as the first female mouse model of late-life social isolation, and provide evidence to inform the development of interventions aimed at promoting functional recovery following isolation in late-life.

## Introduction

Given the recent social isolation recommendations in place globally in response to the COVID-19 pandemic, understanding the impact of isolation on behaviour is important for informing interventions that may mitigate the development of mental health disturbances following the isolation period. People experiencing prolonged periods of isolation commonly report feelings of anxiety and depression, post-traumatic stress, anger, loneliness, and boredom [1, 2], high blood pressure and increased expression of inflammatory markers [3], and are at an increased risk of functional decline and early mortality [4, 5]. Understanding how these effects may differentially influence behaviour in older adults is particularly relevant, as this population frequently experiences late-life social isolation, especially in females due to their longer life expectancy [3, 6].

Rodent models of social isolation offer the ability to study the effects of isolation under highly controlled conditions, controlling for age, duration of isolation, housing conditions, and the developmental time point of isolation. Rodents, like humans, are social creatures that thrive in group housing conditions. The literature on the impact of social isolation on affective behaviour in rodents to date has largely been limited to studying the effects of single-housing isolation-induced stress during critical developmental periods in adolescence and early adulthood (see [7] for review). Such studies typically report increases in depressive-like and anxiety-like behaviours [8, 9], although there are differential effects of isolation across sex, with females typically showing less anxiety-like behaviours than males [10]. Early-life isolation also leads to hyperactivity, increased exploratory behaviour, and increased socialization, suggesting that isolation may have an anxiolytic effect in young animals [8, 11–13]. To date, investigations of the effects of late-life social isolation in aged rodents has been limited to an assessment of social approach behaviour in male rats, where animals exhibited only mild social exploration deficits in response to two weeks of late-life isolation, but this same disruption of social behaviour was not observed in rats isolated for four weeks [14]. As the impact of social isolation on other forms of affective behaviour and cognition in aged animals is not well characterized, we investigated cognitive and affective behavioural disturbances and hippocampal neuroinflammatory cytokine expression in response to late-life isolation in 18-month old female mice.

## Materials and methods

Female F1 hybrid C57BL/6J (Jackson Labs) x 129S6/SvEvTac (Taconic) served as subjects. Mice were bred in the mouse vivarium at Baylor University. Post-weaning, mice were group-housed (3–5 per cage) in standard shoebox cages with bedding and nesting materials, located in ventilated racks in the rodent vivarium. Throughout the study, mice had *ad libitum* access to food and water, and were maintained on a standard 12hr light-dark cycle (lights on 600hr– 1800hr). Mice were weighed at the beginning of the rearing period and checked daily for visual signs of distress. Mice assigned to the isolation condition (*M* = 32.705, *SD* = 8.165) and the group-housed (*M* = 36.110, *SD* = 8.983) condition did not significantly differ in weight at the beginning of the study (*t*_(38)_ = 1.254, *p* = 0.217, *d* = 0.397). To minimize isolation disruption, mice were not weighed on a weekly basis during the rearing period. Any mouse observed exhibiting signs of distress during the rearing period were weighed, and if the distressed mouse lost 20% of its starting body weight, the mouse would have been excluded from the study (n = 0). All behavioural tests were conducted between 900hr– 1700hr.

**Fig 1:**
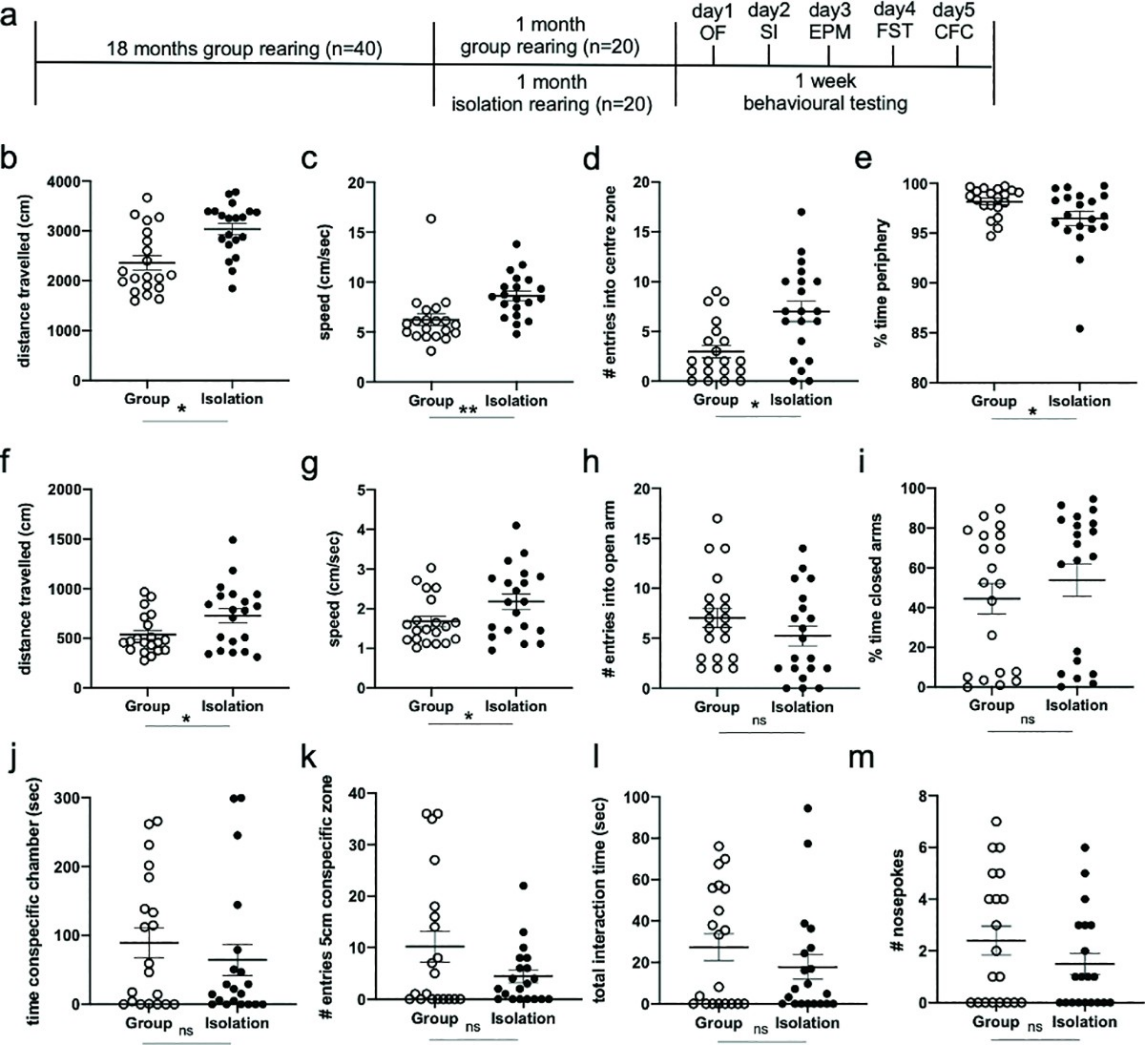
Late-life social isolation increases exploration and hyperactivity in female mice. (a) Experimental timeline and behavioural test-order. *OF* = open field, *SI* = 3-chambered social interaction task, *EPM* = elevated plus maze, *FST* = forced swim task, *CFC* = context fear conditioning. In the open field task, (b) isolated mice (black, closed circles) travel further distance, (c) move at a faster speed, (d) make more entries into the center of the open field arena, and (e) spend less time in the periphery of the arena than group-housed controls (white, open circles), indicating a less anxiety-like phenotype. In the elevated plus maze, (f) isolated mice travel further distance and (g) move at a faster speed, but (h) make a comparable number of entries into the open arms of the maze, and (i) spend an equivalent amount of time in the closed arms of the elevated plus maze as controls. In the social interaction task, (j) isolated and group-housed control mice spend an equivalent amount of time in the chamber containing the conspecific, (k) make a similar number of entries into the zone surrounding the conspecific’s cage, (l) interact for a comparable length of time with the conspecific, and (m) make a similar number of nose pokes into the conspecific’s cage. Error bars represent the standard error of the mean (SEM). * p < 0.05, ** p < 0.01, ns p > 0.05.

At 18 months of age, home cages of mice were randomly divided into a group-housed condition (n = 20 mice) and a single-housed social isolation condition (n = 20 mice). Eighteen months was selected as a time point for late-life isolation, in line with previous investigations modeling depressive-like behaviour and cognitive impairment in aged mice [15, 16]. For subjects assigned to the social isolation condition, each mouse was individually housed in a standard shoebox cage with bedding and nesting materials, *ad libitum* access to food and water, in ventilated racks in the rodent vivarium for one month. Isolated mice were unable to see mice in neighboring cages. Group-housed mice were housed in a total of five cages, with the same cage-mates throughout the entire rearing period. At the end of the one-month isolation period, mice remained in their assigned housing conditions and began a post-rearing behavioural test battery to assess anxiety-like and depressive-like behaviour and contextual fear memory (see Fig 1A for experimental timeline).

Mice were handled individually by an experimenter for 5 min/day for 5 days prior to beginning behavioural testing. All behavioural testing was conducted by DGS and KG. For each task, all mice were tested by the same experimenter. Behavioural tasks were conducted in a least-to-most aversive order (see Fig 1A for test order), and each test apparatus was cleaned with 70% ethanol between trials. Only a single behavioural task was conducted on each day. Mice were returned to their homecage following completion of each behavioural task. *Open Field (OF)*: Mice were placed individually in the open field arena (45 cm x 45 cm x 40 cm, white, solid acrylic walls, Panlab) for 15 min. The percentage of time spent in the different areas (periphery, middle zone, and center zone) of the open field, and the distance and speed travelled were analyzed. The periphery is defined as the area within 10 cm from the edge of the wall. The middle zone is defined as the area 5 cm between the periphery and center zone. The center zone is the 15 cm^2^ area within the middle of the arena. Preference for the periphery of the arena is indicative of an anxiety-like phenotype. *3-Chambered Social Interaction Task (SI)*: The social interaction arena consists of three equally sized clear acrylic chambers (43 cm x 20 cm x 23 cm each chamber, Panlab) connected by a sliding door on either side of the center chamber. Open air isolation cages (8 cm diameter, 18 cm height, metal bars spaced 6 mm apart) were located in each of the side chambers. One isolation cage contained a novel sex-matched conspecific. The other cage remained empty during the trial. The test mouse was placed in the center chamber and allowed 5 min to habituate to the chamber. The sliding doors were then raised, the mouse was allowed to freely explore all three chambers of the arena for an additional 5 min. The duration the test mouse spends facing the conspecific’s cage with its nose within 1 cm of the cage (total interaction time), and the number of nose pokes emitted by the test mouse between two bars of the conspecific’s cage were manually scored by an experimenter blind to condition. The number of entries into a 5 cm zone surrounding the conspecific cage, and the time spent exploring the chamber containing the conspecific relative to the other two chambers were measures using video-tracking software. Increased time spent in the social side chamber relative to the non-social side chamber indicates a preference for social interaction. *Elevated Plus Maze (EPM)*: The white Plexiglas apparatus, constructed in the Baylor University machine shop, consisted of two open arms (30 cm long × 5 cm wide × 0 cm high) and two closed arms (30 cm long × 5 cm wide × 15 cm high) extended from a central platform (5 cm x 5 cm) elevated 50 cm above the floor. Mice were individually placed on the central platform facing an open arm and allowed to freely explore the maze for 5 min. The number of entries (all 4 paws) into open and closed arms of the maze, and the total time spent in open and closed arms of the maze were measured. *Forced Swim Task (FST)*: A clear Plexiglas cylinder (10 cm diameter, 25 cm high, Panlab) was filled halfway with room temperature water. The mouse was placed in the chamber for 6 min. After completion of the trial, mice were pat dried with a towel and placed back in the home cage. The latency to immobility, and the durations of immobility and high activity during the task was calculated. High activity was defined by the SMART video-tracking software as ≥ 15% change in pixels between serial video frames during the task. Lower periods of activity are indicative of a learned helplessness phenotype or depressive-like behaviour. *Context Fear Conditioning and Testing (CFC)*: Fear conditioning was conducted in a chamber (19 cm × 20 cm × 128 cm) with a shock-grid floors (bars 3.2 mm in diameter spaced 7.9 mm apart), clear acrylic front and back walls, and aluminum side-walls and roof (Coulbourn Instruments). Mice were allowed 2 min to explore the chamber, then received 3 foot shocks (0.5 mA, 2 sec duration, 1 min apart). Mice were removed from the chamber 1 min after the last shock. Twenty-four hours later, the mouse was replaced in the conditioning chamber and freezing behaviour was recorded for 3 min. Once all mice from the home cage completed the fear conditioning trial, all mice were returned to the home cage. Mice were temporarily held in a shoebox cage with standard bedding between the fear conditioning trial and their return to the home cage (no more than 20 min). Freezing is a species-specific defense reaction that is typically used as a measure of fear in rodents. Behaviour in the chamber was recorded by an overhead camera, and activity levels were analyzed using Freezeframe software (Actimetrix, RRID:SCR_014429).

mRNA expression of brain derived neurotrophic factor (*Bdnf*) and markers of neuroinflammation (*Il-6*, *Il-1β*, *Tnf-α*), and the upregulation of microglia surface antigens (*Itgam*) were assessed in the hippocampus. Following behavioural testing, a subset of mice (n = 8 per group) were euthanized via rapid decapitation, the hippocampus was rapidly dissected, and stored at −80°C until processed. RNA was extracted using the E.Z.N.A. RNA Isolation Kit II (Omega BioTek, Norcross, GA). cDNA was transcribed using the High Capacity cDNA Reverse Transcription Kit (Applied Biosystems by Life Technologies, Grand Island, NY), and quantitative real-time protein chain reaction (qPCR) was carried out in a QuantStudio 6 PCR machine using TaqMan gene expression assays (Applied Biosystems by Life Technologies, Grand Island, NY). Targets analyzed included: *Il-6* (cat number: Mm00446190_m1; Applied Biosystems), *Il-1β* (cat number: Mm00434228_m1; Applied Biosystems), *Tnf-α* (cat number: Mm00443258_m1; Applied Biosystems), *Bdnf* (cat number: Mm04230607_s1; Applied Biosystems), and *Itgam* (cat number: Mm01271259_g1; Applied Biosystems). *Gapdh* (cat number: Mm99999915_g1; Applied Biosystems) served as the endogenous internal control. Reactions were performed in duplicate with the fold difference for each gene calculated using the 2−ΔΔCT method. One brain from the social isolation group was excluded from qPCR analyses due to poor tissue integrity. Two brains from the group-housed group were excluded from *Il-6* analyses due to a processing error.

Activity during other behavioural tasks was recorded by a digital camera and activity levels were analyzed using the SMART video-tracking system software (Panlab, RRID: SCR_002852). Independent samples t-tests (2-tailed) were conducted for all behavioural tasks and qPCR analyses, and effect size (Cohen’s d) are reported. All behavioural statistical analyses were conducted using SPSS 26 (RRID: SCR_002865) by an experimenter blind to experimental condition. Effects were considered significant at p < 0.05. Where appropriate, Bonferroni corrections for multiple comparisons were applied to t-tests. Figures depicting ± standard error of the mean (SEM), as well as all individual subject’s data points. All procedures were approved by Baylor University’s Institutional Care and Use Committee and conducted in accordance with the Guide for the Care and Use of Laboratory Animals from the National Institutes of Health.

## Results

Socially isolated mice engaged in more exploratory behaviour in the open field task, travelling a further distance during the open field trial (*t*_(38)_ = -3.70, *p* = 0.001, *d* = 1.169; Fig 1B), and at a faster speed (*t*_(38)_ = -3.09, *p* = 0.004, *d* = 0.972; Fig 1C), than group-housed control mice. Isolated mice displayed a slightly less anxious phenotype than group-housed mice, making more entries into the center zone of the arena (*t*_(38)_ = -3.33, *p* = 0.002, *d* = 1.054; Fig 1E) and spending less time in the periphery of the arena (*t*_(38)_ = 2.15, *p* = 0.038, *d* = 0.681; Fig 1E), although this did effect not survive the correction for multiple comparisons for the open field. Similar to the open field task, during the elevated plus maze task, isolated mice explored significantly further distances than group-housed controls (*t*_(38)_ = -2.29, *p* = 0.028, *d* = 0.723; Fig 1F), and trending towards activity at a greater speed than controls (*t*_(38)_ = -2.12, *p* = 0.041, *d* = 0.700; Fig 1G). Unlike the open field, however, isolated mice did not exhibit reduced anxiety-like behaviour relative to group-housed mice, with mice in both conditions making a comparable number of entries into the open arms of the arena (*t*_(38)_ = 1.30, *p* = 0.201, *d* = 0.412; Fig 1H). The mean percentage of time spent in the in closed arms was not significantly different between group-housed and isolated mice (*t*_(38)_ = -0.85, *p* = 0.403, *d* = 0.268; Fig 1I), suggesting that, despite being more exploratory and hyperactive, late-life isolation did not strongly affect anxiety-like behaviour in the elevated plus maze.

During the social interaction task, no significant differences between isolated and group-housed mice were observed for any of the measures of social exploration behaviour. Mice in both conditions spent comparable time in the chamber containing the conspecific interaction mouse (*t*_(38)_ = 0.80, *p* = 0.427, *d* = 0.254; Fig 1J), comparable time interacting within 5 cm of the conspecific’s cage (*t*_(38)_ = 1.08, *p* = 0.285, *d* = 0.343; Fig 1L), as well as a similar number of entries into the 5 cm zone surrounding the conspecific’s cage (*t*_(38)_ = 1.78, *p* = 0.084, *d* = 0.343; Fig 1K), and number of nose pokes (*t*_(38)_ = 1.29, *p* = 0.206, *d* = 0.407; Fig 1M), suggesting that the socially isolated mice exhibited neither a preference for, nor an aversion to, social proximity and exploration when given the opportunity to interact with a novel conspecific.

**Fig 2:**
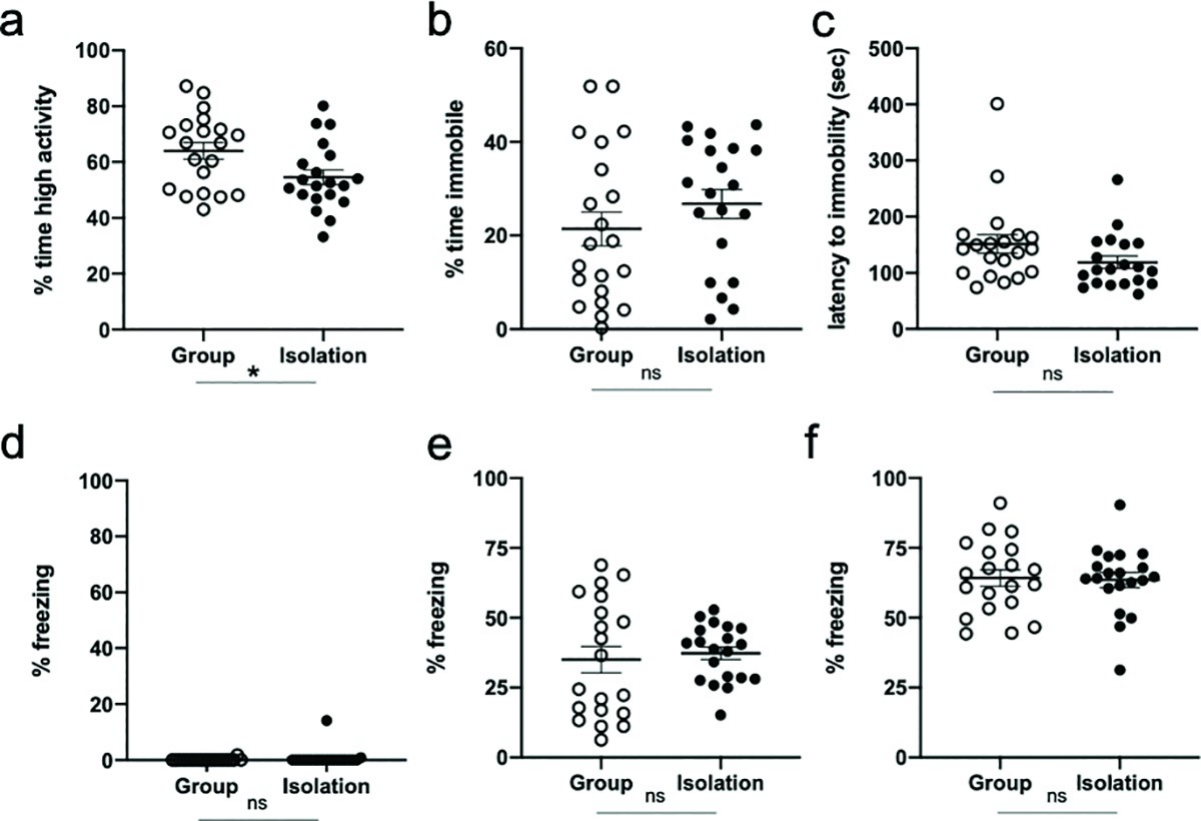
Late-life social isolation does not enhance depressive-like behaviour or context fear behaviour. (a) Socially isolated mice (black, closed circles) spend less time performing high-activity struggling behaviour in the forced swim task relative to group-housed controls (white, open circles), indicating a mild learned-helplessness phenotype. (b) Isolated and group-housed mice spend a similar percentage of time immobile, and (c) similar latency to the first bout of immobility in the forced swim task. (d) Isolated and group-housed controls exhibit comparable freezing behaviour pre-foot shocks, and (e) post-foot shocks during context fear conditioning. (f) Freezing during the context fear test session was equivalent between isolated and group-housed mice, indicating no enhanced fear reactivity or context fear memory following late-life social isolation. Error bars represent the standard error of the mean (SEM). * p<0.05, ns p > 0.05.

Isolated mice displayed a mild learned helplessness phenotype during the inescapable forced swim task, spending less time performing high activity struggling during the task relative to group-housed controls (*t*_(38)_ = 2.37, *p* = 0.023, *d* = 0.749; Fig 2A). No difference in behaviour was observed between conditions in the percentage of time spent immobile during the task (*t*_(38)_ = -1.49, *p* = 0.146, *d* = 0.582; Fig 2B) and the latency to immobility (*t*_(38)_ = 1.65, *p* = 0.106, *d* = 0.523; Fig 2C), suggesting that late-life isolation did not induce a robust depressive-like phenotype.

Context fear conditioning is typically used as a measure of context-dependent memory, but also assesses fear and anxiety-like behaviour [17–19]. Prior to the presentation of shock, both group-housed and socially isolated mice exhibited comparable levels of activity in the conditioning chamber (*t*_(38)_ = -0.93, *p* = 0.357, *d* = 0.295; Fig 2D), indicating that late-life isolation alone did not contribute to a baseline increase in anxiety-like behaviour during the task. Following the presentation of the three foot shocks, both groups exhibited equivalent levels of post-shock freezing during the conditioning trial (*t*_(38)_ = -0.44, *p* = 0.665, *d* = 0.138; Fig 2E). Twenty-four hours later, both groups exhibited comparable levels of freezing during the context memory test (*t*_(38)_ = 0.18, *p* = 0.859, *d* = 0.057; Fig 2F). Together these findings indicate that late-life social isolation did not impair the mouse’s ability to form a context fear memory, nor did it enhance the sensitivity or reactivity of the mice to an aversive shock stimulus.

**Fig 3:**
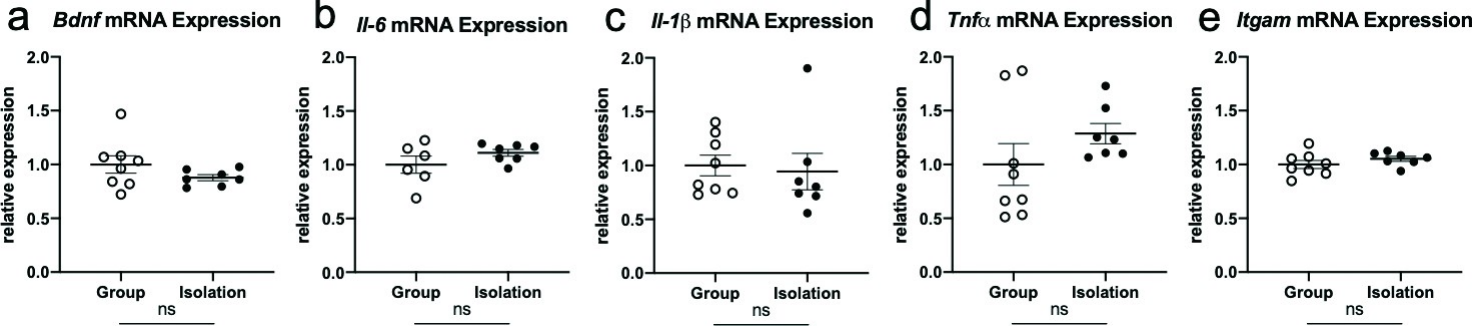
Late-life social isolation does not alter mRNA expression of inflammatory cytokines or microglial activation. No significant differences in hippocampal mRNA expression levels of (a) *Bdnf*, (b) *Il-6*, (c) *Il-1β*, (d) *Tnf-α*, or (e) *Itgam* in socially isolated mice (black, closed circles) relative to group-housed controls (white, open circles).

Alterations in inflammatory cytokines (*IL-6*, *IL-1β*, *Tnf-α)* or microglial activation *(Itgam)*, and growth factors expression (*Bdnf*) in response to late-life isolation was assessed within the hippocampus. qPCR analyses revealed a non-significant trend towards increased mRNA expression of *Il-6* and *Tnf-α* in isolated mice relative to group-housed mice (*Il-6* (t_(11)_ = 1.37, p = 0.199, d = 0.736, Fig 3B), *Tnf-α* (t_(13)_ = 1.27, p = 0.228, d = 0.671, Fig 3D). No group differences were observed for the expression levels of *Bdnf* (t_(13)_ = -1.34, p = 0.202, d = 0.717, Fig 3A), *Il-1β* (t_(13)_ = -0.30, p = 0.769, d = 0.152, Fig 3C), and *Itgam* (t_(13)_ = 1.15, p = 0.271, d = 0.605, Fig 3E). These results indicate that one month of late-life isolation is not sufficient to cause a significant change in neurotrophic growth factor, neuroinflammatory cytokines, or microglial activation in aged females.

## Discussion

To date, limited studies have assessed the impact of social isolation in aged rodents, with only two studies, to our knowledge, having assessed social behaviour following at least two weeks of late-life isolation in aged rodents [14, 20]. Here, we found that month-long late-life isolation increased hyperactivity and exploratory behaviour, while not significantly affecting normal social interaction behaviour, depressive-like behaviour, or fear learning and memory in aged female mice. It is important to note that while including only female subjects limits the interpretations one can make based on sex, the characterization of the impact of late-life isolation in female subjects contributes to the literature by addressing a question that, to date, has been explored in studies using exclusively male animals. Our results are consistent with those observed following four weeks of isolation in male rats, where late-life isolation did not impair social approach behaviour relative to grouped controls [14]. Our results differ from that of Wang et al. [20] who report that male mice following eight weeks of social isolation spend significantly less time in the same chamber as a novel conspecific relative to group-housed mice. This discrepancy may be indicative of sex differences in interaction behaviour following late-life isolation, or may be influenced by the extended period of isolation used in their study. Our results further add to the gap in the literature on social isolation in female mice by characterizing affective behaviour in response to late-life isolation. The results also complement findings in young mice, suggesting that hyperactivity induced by social isolation is observed across the lifespan [11–13].

Specifically, isolated mice exhibited increased hyperactivity and exploratory behaviours in the elevated plus maze and open field tests relative to controls. Social isolation studies in adolescent or young-adult rodents similarly find that isolation increases exploration and locomotor activity and reduces anxiety-like behaviour in these tasks [11, 13, 21]. Though speculative, observed hyperactivity and exploratory behaviours in novel contexts may, in part, be a result of declines in inhibitory GABA-A receptors in the hippocampus and cortex following isolation [22]. Young male mice exhibit stronger exploratory behaviour in the elevated plus maze than females following isolation [23], however further studies are needed to determine if these sex differences would be preserved following late-life isolation. Isolated aged mice also displayed less anxiety-like behaviour in the open field, travelling into the center of the arena more than group-housed animals and spending less time in the periphery of the maze, although this same risk-taking behaviour was not observed in the elevated plus maze, suggesting that isolation has only a mild anxiolytic effect in aged mice that may decline over repeated behavioural exploration sessions. Previous studies have reported decreased [23, 24] and comparable [25] anxiety-like behaviours in isolated young mice in this exploratory task.

Hyperactivity was not observed in an inescapable swim task. Isolated mice showed moderate behavioural despair, as indicated by less time spent performing high-activity swimming during the forced swim task relative to controls, but no difference in immobility behaviour was observed between groups, suggesting that isolation did not induce a robust depressive-like phenotype. The effects of isolation on depressive-like symptoms in young mice are mixed, with reports of more immobility particularly in males [8, 26, 27], while others find less [28], or no difference in immobility [21] behaviour between single and group-housed animals.

Interestingly, isolation did not mediate fear behaviour, nor preference for social engagement. The impact of isolation on subsequent social behaviour in young rodents is mixed, where male mice isolated in young adulthood have displayed increased social interaction behaviour [8]. While male rats isolated after weaning displayed reduced interactions and increased freezing behaviour [29]. The lack of potentiation of a hippocampal-dependent context fear memory in aged mice is consistent with cellular investigations finding no difference in the electrophysiological properties of CA1 and CA3 following month-long isolation in young mice [11] as well as our own findings of unaltered expressions of hippocampal *Bdnf*, and no evidence of enhanced neuroinflammatory or microglial activation in the hippocampus in response to the same isolation duration. Wang et al. [20] demonstrated that two months of social isolation in aged male mice is sufficient to alter *Bdnf*, *IL-6*, *IL-1β*, and *Tnf-α* expression levels, which they found can be largely prevented by environmental enrichment during isolation. A recent study of isolation in juvenile mice found reduced dendritic arborization and dendritic spine density of hippocampal CA1 neurons [27], suggesting that isolation stress may structurally remodel the hippocampus, a brain region critical for memory formation and storage [30]. Liu et al. al [27] also found suppressed levels of plasticity-related proteins crucial for memory formation, including BDNF, phosphorylated CREB, and synaptic proteins synaptophysin and PSD95 in both young males and females following social isolation rearing. In the amygdala, a region involved in regulating emotional and social behaviour, early-life isolation in male rats was associated with in-vivo electrophysiological hypoactivity and reduced volume of the amygdala’s medial nucleus [31, 32]. These robust findings suggest that affective behavioural and cognitive disruptions associated with early-life isolation may be mediated by underlying suppression of neural plasticity in regions of the limbic system, including the hippocampus and amygdala, that are critical for normal affective regulation and memory. Further studies are required identify other physiological mechanisms that may be disrupted following isolation in the aged brain, how the degree of disruption may differ by sex, and how the duration of isolation may produce differing effects across the lifespan.

In summary, late-life social isolation in aged female mice did not lead to depressive-like symptomology, altered social interaction behaviour, sensitivity to context fear acquisition and memory, or alterations in hippocampal inflammatory cytokines. Rather, isolation increased hyperactivity and exploration behaviours, findings that have been similarly observed in both sexes following early-life isolation [8, 11–13], although an early-life time point was not assessed directly in this study which limits the direct comparisons that can be made across sex and age. One caveat to note is that the limited group differences in cognitive and affective behaviour, and neuroinflammation and microglial activation may be, in part, due to the stress induced by multiple behavioural testing sessions in both groups.

The observed hyperactivity and exploration in aged female mice are consistent with observations in older adults who lose social connections, through widowhood, for example, may seek greater involvement in social activities and volunteering [33]. While younger adults who experience loneliness seem to preferentially attend to social threats, older adults focus attention on positive experiences, such as emotional closeness with loved ones [34]. Neuroimaging research demonstrates differential ventral striatum activity in response to photos of strangers vs. close others, suggestive of social “craving” found in those experiencing perceived social isolation [35]. Humans, like mice, may engage in enhanced novelty seeking exploratory behaviour with the goal of building new connections with close others in their social networks, which may be protective against perceived loneliness and subsequent cognitive decline and psychological distress [33, 36–40]. These findings have substantial value by identifying potential disturbances in cognition and neuroinflammation following isolation in an animal model that may be used to inform the development of therapeutic interventions in older adults. Furthermore, these findings highlight the need for further studies comparing the behavioural and neural impact of social isolation across the lifespan, in both sexes, and for varying durations, as the sensitivity to developing deleterious effects may differ across the lifespan. Predicting disturbances in mental health in response to prolonged isolation will allow clinicians and community health leaders to employ evidence-based prevention programs to mitigate the risk of mental illness [41].

## Supporting information

S1 Data (XLSX)

S2 Data (XLS)
